# Executives’ ESG cognition and enterprise green innovation: Evidence based on executives’ personal microblogs

**DOI:** 10.3389/fpsyg.2022.1053105

**Published:** 2022-12-05

**Authors:** Deli Wang, Yonggen Luo, Shiyang Hu, Qi Yang

**Affiliations:** ^1^School of Accounting, Guangdong University of Foreign Studies, Guangzhou, China; ^2^Research Center for Guangdong-Hong Kong-Macao Greater Bay Area Accounting and Economic Development, Guangdong University of Foreign Studies, Guangzhou, China; ^3^School of Accounting, Guangdong University of Finance and Economics, Guangzhou, China; ^4^School of Economics and Business Administration, Chongqing University, Chongqing, China

**Keywords:** ESG cognition, green innovation, microblogs, China, text analysis

## Abstract

Based on cognitive theory, we investigated the influence of executives’ ESG cognition on corporate green innovation using data from Chinese manufacturing listed companies from 2010 to 2019. The paper first constructs a metric of ESG cognition of company executives by presenting a quantitative analysis of data from their personal microblogs using textual analysis. The findings show that executive ESG perceptions significantly improve corporate green innovation. After addressing the endogeneity issue through a series of robustness tests, the findings of this paper still held true. Further research found that the enhancement effect of executive ESG perceptions on firms’ green innovation level was mainly found in the sample without heavy pollution and with lower financing constraints and a higher marketization process. This study makes an important contribution to the research on corporate green innovation based on the perspective of executive ESG cognition while also providing a theoretical basis and practical reference for corporate green innovation practices.

## Introduction

Cognition is an important psychological concept; it is an individual’s ability to perceive, reason about, and construct ideas in response to environmental and organizational stimuli and is the basis for decision-making and behavior (Bandura, 1989; Beauchamp et al., 2019; Cristofaro, 2020; Schunk and DiBenedetto, 2020). Cognitive theory suggests that human behavior is the product of the interaction between the self-system and the external environment (Wood and Bandura, 1989; Bandura, 1991). Executives play a decisive role in the organization of the firm. In fact, since the introduction of the concept of limited rationality (Simon, 1955), executive perception has been an important topic of academic debate. A rich body of literature has explored the crucial influence of corporate executives’ perceptions from the perspectives of firm performance, investment decisions, cash holdings, corporate social responsibility, and surplus management (Orens and Reheul, 2013; Chen et al., 2014; Li et al., 2020; Sarfraz et al., 2020; She et al., 2021; Zhang et al., 2021; Berthet, 2022). However, little work has focused on the economic consequences of executives’ ESG perceptions.

ESG is gradually being taken seriously by the practical and academic communities as social responsibility issues such as climate risk, environmental pollution, and financial fraud are becoming increasingly serious. Especially after the inclusion of the MSCI index system, ESG is of great importance for Chinese enterprises, who can use it to enhance their competitive advantage (Albuquerque et al., 2019). As the standard bearers of social and economic development, enterprises are the subjects of ESG practice. According to cognitive theory, human behavior is influenced by the observation and interpretation of the environment during the learning process, and cognition, behavior, and environment are interdependent causal structures (Wood and Bandura, 1989). Therefore, changes in the external environment brought about by ESG and its related policies will also cause changes in the cognition of corporate executives, which in turn will affect micro-firm behavior. Green innovation, as a key way to promote green transformation, is highly dependent on the perceptions of corporate executives and their investment decisions. As green innovation is characterized by high investment, high risk, and long investment return cycles, it is often not prioritized in terms of resources and capabilities (Aguilera-Caracuel and Ortiz-de-Mandojana, 2013; Huang and Li, 2017). The willingness of executives to use organizational resources for green innovation depends on their perceptions and interpretations of environmental protection. Huang et al. (2019) found that executives’ environmental awareness had a positive impact on their firms’ technological innovation, and executives with good environmental awareness would invest more in research and development (R&D), which would help firms achieve higher technological innovation efficiency. Therefore, the ESG awareness of executives can provide resources and technology to ensure the green innovation behavior of enterprises and thus improve their level of green innovation.

In view of this, we used cognitive theory and a sample of Chinese manufacturing listed companies to investigate the impact of executives’ ESG cognition on corporate green innovation. First, we conducted a text analysis of data crawled from the personal microblogs of corporate executives and used the data to construct quantitative indicators of ESG cognition of corporate executives. Second, based on the indicators of executive ESG perceptions, we empirically examined the impact and mechanism of executive ESG perceptions on corporate green innovation.

This paper makes several contributions. First, using cognitive psychology theory, our research further enriches and extends the study of the economic consequences of cognitive theory on corporate innovation behavior by incorporating corporate executives’ cognitive factors into the study of corporate green innovation. Previous studies have focused on the impact of ESG ratings on corporate green innovation (Zheng et al., 2022) while also noting the important role of executive cognition (Mo et al., 2022; Tan and Zhu, 2022). In contrast to previous studies, this paper focuses on the influence and mechanisms of executive ESG cognition on corporate green innovation behavior based on a cognitive theoretical framework, which further enriches the research on executive cognition. Second, this paper explores the influence of executives’ ESG cognition on corporate green innovation behavior, which further enriches the research on the factors influencing corporate green innovation. Previous studies on firms’ green behavioral decisions have mainly focused on the influence of government environmental regulations, market demand, public pressure, and firms’ profitability (Fernando and Wah, 2017; Chen et al., 2018; Chen and Liu, 2020; Wang et al., 2021; Gao et al., 2022; Xu et al., 2022), but these studies have neglected the subjective initiative of firm management. This paper, however, explores the motivation of corporate green innovation from the perspective of managers’ cognition, further enhancing the research on the factors influencing corporate green innovation behavior.

## Literature review

### Executive cognition

Since the introduction of cognitive theory, a rich body of literature has explored the composition of executive cognition and its economic consequences for business organizations from different perspectives. Early on, scholars attempted to analyze the composition and characteristics of executive cognition, thus introducing several important concepts such as attention (Cho and Hambrick, 2006; Cristofaro, 2020; Schunk and DiBenedetto, 2020), selective perception (Sutcliffe and Huber, 1998), and blind spots (Zajac and Bazerman, 1991). In subsequent studies, using textual analysis, scholars have built on this to analyze the factors influencing the cognitive complexity of executives (Graf-Vlachy et al., 2020). One study by Eftekhar et al. (2014) analyzed the link between Facebook users’ photo-related activities and the “big five” personality traits and found that neuroticism and extraversion, among other connections, predicted more photo uploads. Obschonka and Fisch (2018) analyzed Trump’s personality traits by means of computer linguistic text analysis using content posted by him on Twitter. Scholars have also focused on the impact that company executives’ perceptions have on corporate organizational behavior and found that executives’ perceptions can significantly influence firm performance (Kim et al., 2016; Ou et al., 2018; Park et al., 2018). The economic consequences of specific executive cognitions have also been examined; for example, Liu and Xi (2021) found that CEO entrepreneurial cognitive orientation affects firm performance by triggering middle managers’ confidence in organizational prospects or workplace anxiety.

### Green innovation

Green innovation refers to activities such as developing new or improving existing product designs, processes, and organizational management in order to achieve the sustainable development goals of both economic and environmental benefits (Chen, 2008; Wang et al., 2021; Xu et al., 2022). Green innovation can be divided into two categories: Green product innovation and green process innovation (Gunasekaran and Spalanzani, 2012). The current research on green innovation focuses on its drivers and performance evaluation. Regarding the drivers of green innovation, there is a large body of literature using institutional and stakeholder theories to explore the impact of environmental regulatory pressure and stakeholder pressure on corporate green innovation from the perspective of the external environment (Delgado-Ceballos et al., 2012; Fernando and Wah, 2017; Chen et al., 2018; Chen and Liu, 2020). Based on the internal environment perspective, the driving role of organizational resources and capabilities and managers’ perceptions is explored using resource-based theory (Lin and Chen, 2017; Qiu et al., 2020; Singh et al., 2020). There is also literature discussing the relationship between green innovation and firm performance. However, there is still academic disagreement on whether green innovation can improve firm performance. Green innovation can increase environmental investments, leading to additional costs for firms and consumption of their limited resources, which has a negative impact on firm performance (Aguilera-Caracuel and Ortiz-de-Mandojana, 2013; Huang and Li, 2017). However, several studies have also argued that green innovation can improve a firm’s green competitiveness and competitive advantage and enhance firm performance (Porter and Van der Linde, 1995; Hojnik and Ruzzier, 2016; Gao et al., 2022).

In general, although there is much discussion of executive cognition and corporate green innovation in the literature, there are still several shortcomings. First, existing studies on green innovation discuss more external factors, such as environmental regulation and stakeholder pressure, and largely ignore the influence of subjective factors of executive cognition. Second, previous studies on executives’ environmental perceptions have mostly used cross-sectional data *via* questionnaires, while fewer have used medium-and long-term panel data from listed companies to verify their findings, limiting the relevance. Therefore, we used cognitive theory to examine the impact of executive ESG on green innovation.

### Executive cognition and green innovation

The perceptions of corporate executives play an important role in influencing organizational behavioral decisions and economic performance and are a key element in explaining the different ways in which organizations respond to institutional pressures in the same environment (Hambrick, 2007). As decision-makers in a firm’s innovation strategy, the way executives identify and interpret opportunities and challenges in the external environment will be reflected in the firm’s behavioral decisions, i.e., executives’ perceptions intervene and determine the firm’s behavioral choices.

Executive ESG cognition refers to the cognitive structure and cognitive process by which corporate executives pay attention to, interpret, and judge ESG policy information in the face of a complex internal and external environment and apply it to corporate decision-making. This paper argues that executive ESG cognition has an important driving role in corporate green innovation behavior. First, due to the huge costs associated with green manufacturing, companies are increasingly aware of the need to break the inherent production model through innovation. The stronger the ESG perception of corporate executives, the more likely they are to gain original and unique leadership through green technology innovation behaviors, achieving a win-win situation for both the economy and the environment. Liu et al. (2012) found that the environmental management practices of companies are closely related to the support of top management. Zhang et al. (2015) concluded that the commitment of managers to environmental sustainability has a significant impact on the environmental strategy activities of companies. Second, in the current situation of increasingly stringent government regulations, the ESG perceptions of corporate executives will prompt them to interpret external environmental regulations and stakeholders’ green needs as opportunities for corporate development. They will also be encouraged to take the initiative to assume corporate environmental responsibility, seize the opportunity to transform green innovation into market value, actively allocate organizational resources to fit corporate environmental strategies, and shift corporate production and operations to a green mode of development. This will enable the company to shift its production and operations to a greener model in order to meet the green expectations of its stakeholders and gain a sustainable green competitive advantage. It is evident that corporate green behavior is directly driven by executives’ ESG perceptions. Based on the above analysis, this study argues that the higher the level of ESG perceptions of executives, the more effectively green innovation can be carried out by enterprises. Therefore, we propose the following hypothesis:

*Hypothesis 1*: The level of ESG cognition among executives is positively related to the level of green innovation in companies.

## Research design

### Data

An initial sample was collected of listed companies in China’s manufacturing sector from 2010 to 2019. Company-related data were obtained from the CSMAR database and the CNRDS database. The data related to executives’ ESG perceptions were selected from the microblogs of certified executives on Sina Weibo. The tweets of the executives were crawled by a Python crawler, and a textual analysis of the tweets was then carried out. The Latent Dirichlet Allocation (LDA) was used to analyze the topic distribution of the tweets, and a supervised machine learning algorithm was used to filter the tweets of the executives on the topic of social responsibility. The subsequent analyses in this paper were based on the social responsibility-related tweets posted by executives, and the number of tweets was used to investigate the influence between executives’ perceptions and the level of corporate green innovation.

The initial sample was screened as follows: (1) financial companies were excluded; (2) companies labelled as “ST” and “*ST” were excluded; (3) companies with incomplete data and whose executives did not operate microblogs were excluded. A total of 2,640 observations were retained after the above screening.

### Variables

This section introduces the dependent variable, explanatory variable, and control variables. The names and definitions of the main variables are shown in Table 1.

**Table 1:** Variable definition table.

Variables	Definition
*Green_Innovation*	Green invention and utility model patents granted in the year are added together, plus one for logarithmic value
*Green_Invention*	Green invention patents granted in the year, plus one for logarithmic value
*Green_Utility*	Green utility model patents granted in the current year, plus one to take the log value
*ESG_Ratio*	Disclosure ratio, as number of ESG-related tweets/non-related tweets
*ESG_LN*	Degree of disclosure, logarithmic value of the number of ESG-related tweets plus one
*SOE*	An indicator variable that equals one if a firm is a state-owned enterprise, and zero otherwise
*ROA*	Return on assets
*LEV*	Total liabilities divided by total assets
*Size*	The natural logarithm of the market capitalization of a firm
*AGE*	The natural logarithm of the age of a firm
*Board*	The number of directors sitting on the board
*TopShare*	Shareholding ratio of the largest shareholder (%)
*RD*	Proportion of R&D investment to operating income (%)
*Disclosure*	Stock exchange evaluation grade of information disclosure quality of listed companies

#### Dependent variable

The explained variable is the green innovation level of enterprises. In the process of modern innovation mechanisms, patents and technological innovation are interdependent and inseparable. As an object of state protection for enterprises’ inventions and creations, patents to a certain extent reflect the level of innovation of enterprises, so this paper proposes that green patents can be used to effectively measure the level of green innovation of enterprises. Based on the above analysis, this paper adopts the sum of green invention and green utility model patents obtained by manufacturing enterprises in the same year as an indicator to measure the level of green innovation of enterprises. Considering the national classification of patents as invention patents and utility model patents, among which invention patents have a higher level of technological innovation and utility model patents have a higher correlation with product shape and construction, this paper also explores the degree of influence of the explanatory variables on these two classifications, respectively.

#### Explanatory variable

The explanatory variable is ESG cognitive level of executives. Over the past decade, social media platforms such as Twitter and Weibo have grown in importance as interactive communication tools. Weibo, the largest domestic social media platform in China, is essentially a microblogging service and is very similar to Twitter in terms of its social service model. The two share many similarities, such as a large number of users, a huge amount of user-generated content, continuous and rapid growth, and the same content distribution model and core functions. The nature of social media data, which stores rich textual data about user interactions, can help us understand what users are thinking about a topic or event, enabling systematic analysis of the structure or patterns of activity among individuals or groups (Yang et al., 2021). Research in psychology has also found strong support for the usefulness and validity of integrative language analysis. For example, linguistic analysis has been widely used to study social relations, hierarchy, emotions, mindsets, and psychological traits (Pennebaker et al., 1997; Tausczik and Pennebaker, 2010). There are many microblog users in China, and many corporate executives have opened their own authenticated accounts. The tweets posted by users can reflect their perspectives and the extent to which they follow them. The number of ESG-related microblogs posted on Sina Weibo by executives who have opened microblogs was used as an explanatory variable to better measure the ESG awareness level of executives. To accurately measure executives’ ESG perceptions, the following steps were strictly followed in the text analysis session: (1) Analyzing text collation: The microblogging data of executives from listed manufacturing companies were extracted, and the relevant format was converted for batch capturing of keywords. (2) Determining keywords: We focused on the influence of executives’ ESG cognition on corporate green innovation; therefore, words highly related to ESG were selected to build a thesaurus, such as “ESG,” “corporate social responsibility,” “green innovation,” “environmental protection,” etc. (3) Analysis: Python software was used to crawl the keywords and obtain the word frequency counts of the keywords. (4) Summary: To address the issue of text comparability, the ESG level of executives was measured in terms of two dimensions, including (a) the number of ESG-related tweets (ESG LN) and (b) the ratio of the number of ESG-related tweets to the number of non-related tweets (ESG Ratio).

#### Control variables

This paper selects property right nature (SOE), total assets net profit rate (ROA), asset-liability ratio (LEV), listed company size (SIZE), company age (AGE), board size (Board), the largest shareholder holding ratio (TopShare), R&D investment to operating income ratio (RD), and the performance of listed companies in each year made by Shanghai Stock Exchange and Shenzhen Stock Exchange (Disclosure) as the control variables of this paper. The nature of property rights divides listed companies into state-owned enterprises and private enterprises. If they are state-owned enterprises, they are assigned 1, and if they are private enterprises, they are assigned 0; Net profit margin of total assets refers to the profitability of the company; Asset-liability ratio indicates the ability of a company to repay its debts. In this paper, the size of listed companies, the age of the company and the size of the board of directors are treated by logarithm. In order to make the data better meet the normal distribution, the shareholding ratio of the largest shareholder is divided by 100.

### Model

In order to verify the hypothesis of this paper, that is, the influence of executives’ ESG cognition level on the green innovation level of enterprises, we construct a multiple linear regression model as follows:


(1) $$\begin{array}{c} Green\_Innovation_{i,t}=\alpha_{0}+\alpha_{1}ESG\_Ratio_{i,t}/ESG\_LN_{i,t}\\ +Control_{i,t}+Year+Industry+\varepsilon \end{array}$$

Where *i* represents an individual enterprise, *t* represents a year, $\alpha_{0}$ represents an unpredictable random variable, and $\varepsilon_{i,t}$ is an interference term that changes with time and individuals. The variables in the model are explained as mentioned above.

## Results

### Descriptive statistical analysis

Table 2 is the descriptive statistical results of the main variables in this paper. For the level of green innovation, the median level of innovation of the sample companies was 0, indicating that more than half of the manufacturing companies have a poor level of green innovation. The mean value was 0.534, the standard deviation was 0.950, the maximum value was 6.111, and the minimum value was 0, implying that there are large differences in the level of innovation between different companies. For executives’ ESG cognitive level, the mean values were 0.514 and 2.730 respectively, and the median values were 0.254 and 2.197 respectively, with the mean values being greater than the median; this shows that executives’ ESG cognitive level is generally higher. Where the maximum values were 101.1 and 13.25 respectively, and the minimum values were both 0, with the standard deviation being 2.427 and 2.495, indicating that there are large differences in the cognition of executives in different enterprises. For the control variables, the large standard deviations of SIZE, AGE, Board, and RD imply that there are large differences in size, age, and R&D investment between the sample companies.

**Table 2:** Descriptive statistics.

Variables	Obs	Mean	Std. dev.	Min	P25	Median	P75	Max	Skewness	Kurtosis
*ESG Ratio*	2,355	0.5142	2.4267	0.0000	0.0320	0.2537	0.5000	101.12	32.286	1276.1
*ESG LN*	2,355	2.7296	2.4950	0.0000	0.6931	2.1972	4.1589	13.251	1.2245	4.9086
*Green invention*	2,355	0.2843	0.7125	0.0000	0.0000	0.0000	0.0000	5.0689	3.2073	14.492
*Green utility*	2,355	0.3842	0.7896	0.0000	0.0000	0.0000	0.6931	5.7170	2.6001	10.888
*Green innovation*	2,355	0.5343	0.9502	0.0000	0.0000	0.0000	0.6931	6.1115	2.2061	8.3113
*SOE*	2,355	0.2790	0.4486	0.0000	0.0000	0.0000	1.0000	1.0000	0.9856	1.9714
*ROA*	2,355	0.0517	0.0417	−0.0044	0.0206	0.0414	0.0727	0.3721	1.5187	6.7983
*LEV*	2,355	0.3814	0.1867	0.0174	0.2320	0.3700	0.5173	0.9785	0.2849	2.4190
*SIZE*	2,355	9.5449	0.5318	7.6615	9.1567	9.4568	9.8282	11.555	0.8576	3.6896
*AGE*	2,355	2.6988	0.4105	0.6931	2.4849	2.7726	2.9957	3.9120	−0.9241	4.3429
*Board*	2,355	2.1415	0.1920	1.3863	1.9459	2.1972	2.1972	2.8904	−0.2175	4.2328
*Top1*	2,355	0.3413	0.1406	0.0431	0.2317	0.3190	0.4325	0.8649	0.6035	3.1369
*RD*	2,355	4.4836	3.5872	0.0000	2.6200	3.7300	5.4000	35.220	2.6110	14.601
*Disclosure*	2,355	0.9240	0.2651	0.0000	1.0000	1.0000	1.0000	1.0000	−3.1998	11.239

### Baseline results

Table 3 show the regression results of the model (1). The results show that the coefficient of the proportion of ESG disclosure (ESG_Ratio) in column (1) was 0.0299 and was significant at the 1% statistical level (*t*-value of 4.4358). The regression coefficient of the degree of ESG disclosure by corporate executives (ESG_LN) in column (2) was 0.0259 and was significant at the 5% statistical level (*t*-value of 1.9852). This indicates that after considering the effects of control variables such as the firm’s own characteristics, year, and industry, the higher the proportion of ESG-related disclosure (ESG_Ratio) and degree of disclosure (ESG_LN) in the tweets of corporate executives, the higher the level of corporate green innovation. This also indicates that executive ESG awareness enhances corporate green innovation.

**Table 3:** Executives’ ESG cognition and green innovation.

Variables	(1)	(2)
	Green_Innovation	Green_Innovation
*ESG_Ratio*	0.0299*** (4.4358)	
*ESG_LN*		0.0259** (1.9852)
*SOE*	−0.1282 (−1.4983)	−0.1298 (−1.4880)
*ROA*	1.5150** (2.3126)	1.5254** (2.3109)
*LEV*	0.5609*** (2.9636)	0.5543*** (2.9332)
*SIZE*	0.6564*** (5.2906)	0.6573*** (5.2347)
*AGE*	−0.0298 (−0.3036)	−0.0364 (−0.3685)
*Board*	−0.0042 (−0.0188)	−0.0069 (−0.0309)
*TopShare*	−0.2090 (−0.9079)	−0.1933 (−0.8337)
*RD*	0.0384*** (3.4597)	0.0387*** (3.5078)
*Disclosure*	0.1393* (1.9141)	0.1356* (1.8607)
*Constant*	−6.2085*** (−5.6840)	−6.2577*** (−5.6071)
*Year*	*Control*	*Control*
*Industry*	*Control*	*Control*
*N*	2,355	2,355
*Adj_R^2^*	0.2189	0.2171

The *T* value calculated after misadjustment of clustering standard at company level is in brackets; *, ** and *** are significant at the significance level of 10, 5 and 1%, respectively. The following table is the same.

### Further analysis

Corporate green innovation patents can be specifically classified into green invention patents and green utility model patents. Table 4 reports the regression results of executive ESG perceptions on different types of green innovation patents. The results in columns (1) and (3) of Table 5 show that the regression coefficients for the proportion of executive tweets on ESG disclosure (ESG_Ratio) were 0.0178 and 0.0330 respectively, and both were significant at the 1% test level. The results from columns (2) and (4) show that the regression coefficients of the degree of disclosure of ESG on executive microblogs (ESG_LN) were 0.0205 and 0.0213 respectively, and both were significant at the 5% test level. This also indicates that after considering the effects of control variables such as firm characteristics, year, and industry, executive ESG awareness helps to increase the number of green invention patents and green utility model patents of firms. Further, by increasing the number of green invention and green utility model patents, executive ESG awareness in turn improves the level of green innovation of firms. In summary, the ESG awareness level of executives, i.e., the proportion of ESG disclosure (ESG_Ratio) and the degree of ESG disclosure (ESG_LN) in executive tweets, can, to a certain extent, increase the number of green patents represented by green inventions and green utility models, i.e., it can contribute to the level of green innovation of the company.

**Table 4:** Further analysis.

Variables	(1)	(2)	(3)	(4)
	Green_Invention	Green_Invention	Green utility	Green utility
*ESG Ratio*	0.0178*** (4.8153)		0.0330*** (4.7803)	
*ESG_LN*		0.0205** (1.9898)		0.0213** (2.0036)
*SOE*	−0.0961 (−1.5215)	−0.0964 (−1.5071)	−0.1052 (−1.4607)	−0.1079 (−1.4475)
*ROA*	0.4721 (1.0550)	0.4638 (1.0271)	1.1807** (2.0556)	1.2130** (2.0851)
*LEV*	0.3072** (2.2097)	0.2993** (2.1538)	0.4246*** (2.8742)	0.4228*** (2.8686)
*SIZE*	0.5480*** (4.9842)	0.5468*** (4.9988)	0.4680*** (4.3620)	0.4715*** (4.2538)
*AGE*	0.0362 (0.5435)	0.0310 (0.4650)	−0.0300 (−0.3666)	−0.0353 (−0.4290)
*Board*	−0.0371 (−0.1949)	−0.0387 (−0.2050)	−0.0127 (−0.0715)	−0.0155 (−0.0883)
*Top1*	−0.1771 (−1.0780)	−0.1647 (−0.9978)	−0.2281 (−1.1611)	−0.2152 (−1.0891)
*RD*	0.0320*** (4.0233)	0.0322*** (4.0772)	0.0250*** (3.0715)	0.0253*** (3.1230)
*Disclosure*	0.1137** (2.3796)	0.1100** (2.2949)	0.0754 (1.1921)	0.0736 (1.1610)
*Constant*	−5.2969*** (−5.6727)	−5.3198*** (−5.6915)	−4.2801*** (−4.5696)	−4.3436*** (−4.4330)
*Year*	*Control*	*Control*	*Control*	*Control*
*Industry*	*Control*	*Control*	*Control*	*Control*
*N*	2,355	2,355	2,355	2,355
*Adj_R^2^*	0.1933	0.1941	0.1915	0.1852

The *T* value calculated after misadjustment of clustering standard at company level is in brackets; *, ** and *** are significant at the significance level of 10, 5 and 1%, respectively. The following table is the same.

**Table 5:** Exclude zero value of green innovation.

Variables	(1)	(2)
	Green_Innovation	Green_Innovation
*ESG Ratio*	0.0287*** (6.6521)	
*ESG_LN*		0.0461** (2.3669)
*SOE*	−0.1286 (−0.9500)	−0.1309 (−0.9430)
*ROA*	0.2008 (0.1828)	0.0745 (0.0678)
*LEV*	0.7516** (2.4340)	0.6652** (2.1942)
*SIZE*	0.8046*** (4.5374)	0.8152*** (4.5184)
*AGE*	−0.0624 (−0.3607)	−0.0761 (−0.4344)
*Board*	−0.2278 (−0.6009)	−0.2338 (−0.6181)
*Top1*	−0.6378* (−1.7937)	−0.5973* (−1.6775)
*RD*	0.0435*** (3.3149)	0.0443*** (3.3412)
*Disclosure*	0.2026 (1.4109)	0.1918 (1.2654)
*Constant*	−6.1681*** (−4.3424)	−6.3636*** (−4.3558)
*Year*	*Control*	*Control*
*Industry*	*Control*	*Control*
*N*	803	803
*Adj_R^2^*	0.2470	0.2469

The *T* value calculated after misadjustment of clustering standard at company level is in brackets; *, ** and *** are significant at the significance level of 10, 5 and 1%, respectively. The following table is the same.

## Robustness test

In order to further verify the reliability and robustness of the research results, the following robustness tests were conducted. First, we conducted a small sample test. According to the descriptive statistics in Table 2, we found that there were more cases of zero green innovation patents in enterprises. In order to eliminate the biased estimation brought by this part of the sample, we removed the observations with zero green innovation patents in the robustness test and re-ran the regression. In addition, in testing the impact of executives’ ESG perceptions on different types of green innovation, we also removed observations where the number of green inventions obtained by the firm in the year or the number of green utility models obtained in the year was 0 and re-checked the regression results. Second, a negative binomial regression was used, and since the number of green innovation patents also fit the counting model, the test was switched to a negative binomial regression.

### Small sample

Table 5 demonstrates executive ESG perceptions, represented by the percentage of ESG disclosure (ESG_Ratio) and the degree of disclosure (ESG_LN) of executive tweets after removing the observation that the number of green innovation inventions obtained by the firm in the year was zero. Results show that they were significant at the 1% level and the 5% level, indicating that an increase in executive ESG perceptions can enhance the level of green innovation of the firm.

Table 6 reports the regression results after removing the observations that the number of green inventions and the number of green novelties obtained by the firm in the year were zero, respectively. In columns (1) and (3), the regression coefficients of the proportion of executive twitter ESG disclosure (ESG_Ratio) were 0.010 and 0.029, respectively, and passed the 5 and 1% test levels. This indicates that after removing the sample 0 value, the proportion of ESG disclosure by executive microblogging (ESG_Ratio) is more able to enhance the level of green innovation of the company by increasing the number of green inventions obtained by the company. In column (2), the regression coefficient of the degree of ESG disclosure by executives on Twitter (ESG_LN) was 0.060, which passed the 1% test level, while the result in column (4) was positive despite a slight decrease in significance, a result that also supports the findings above.

**Table 6:** Exclude zero value of green inventions.

Variables	(1)	(2)	(3)	(4)
	Green_Invention	Green_Invention	Green utility	Green utility
*ESG Ratio*	0.0095** (2.1346)		0.0291*** (7.9432)	
*ESG_LN*		0.0602*** (3.5452)		0.0297 (1.6049)
*SOE*	−0.1792 (−1.2507)	−0.1604 (−1.1427)	−0.1912 (−1.4392)	−0.2042 (−1.4558)
*ROA*	−0.2479 (−0.1929)	−0.5512 (−0.4283)	0.6804 (0.6151)	0.6851 (0.6202)
*LEV*	0.6094* (1.8180)	0.4329 (1.3105)	0.5134* (1.7473)	0.4526 (1.5954)
*SIZE*	0.8812*** (5.2232)	0.8766*** (5.2052)	0.6495*** (3.6867)	0.6677*** (3.6075)
*AGE*	−0.1757 (−0.9291)	−0.2012 (−1.0370)	0.0615 (0.3651)	0.0570 (0.3340)
*Board*	−0.2949 (−0.7305)	−0.3267 (−0.8107)	−0.1760 (−0.5053)	−0.1746 (−0.4982)
*Top1*	−0.4547 (−1.2863)	−0.4169 (−1.2020)	−0.4859 (−1.4227)	−0.4492 (−1.3065)
*RD*	0.0379*** (2.7477)	0.0373*** (2.7454)	0.0151 (1.2522)	0.0165 (1.3443)
*Disclosure*	0.2575** (2.4274)	0.2066* (1.8500)	0.1727 (1.1199)	0.1668 (1.0387)
*Constant*	−6.7475*** (−5.1790)	−6.8110*** (−5.5826)	−5.0680*** (−3.5465)	−5.3189*** (−3.4187)
*Year*	*Control*	*Control*	*Control*	*Control*
*Industry*	*Control*	*Control*	*Control*	*Control*
*N*	465	465	629	629
*Adj_R^2^*	0.2757	0.2955	0.2394	0.2273

The *T* value calculated after misadjustment of clustering standard at company level is in brackets; *, ** and *** are significant at the significance level of 10, 5 and 1%, respectively. The following table is the same.

### Negative binomial regression

The data on patent applications without the natural logarithm were discrete variables, and there were a large number of zero values in their distribution, which may not conform to the assumption of normal distribution, and the expectation of the variables was not equal to the variance. Given this, we adopted negative binomial regression to deal with the problem of non-normal distribution of the explained variables.

Table 7 shows that after switching to the regression treatment, the results were still robust, the significance improved, and the regression coefficient was still positive. The results in column (1) show that the regression coefficient of the proportion of ESG disclosure by executives on Weibo (ESG_Ratio) was 0.0058 and was significant at the 5% test level. The results in column (2) show that the regression coefficient of the degree of ESG disclosure by executives on Weibo (ESG_LN) was 0.0408 and was significant at the 5% test level. This result is generally consistent with the results of the previous regression using OLS method and supports the conclusion in the previous paper that the degree of ESG disclosure by executives (ESG_LN) can enhance the level of corporate green innovation.

**Table 7:** Executives’ ESG cognition and green innovation.

Variables	(1)	(2)
	Green_Innovation	Green_Innovation
*ESG Ratio*	0.0058** (2.4406)	
*ESG_LN*		0.0408** (2.1859)
*SOE*	−0.1407 (−1.0543)	−0.1366 (−1.0279)
*ROA*	3.8987*** (3.4436)	3.7868*** (3.3660)
*LEV*	1.3762*** (3.7504)	1.3246*** (3.6518)
*SIZE*	0.8413*** (7.4715)	0.8350*** (7.6571)
*AGE*	−0.1615 (−0.7556)	−0.1844 (−0.8491)
*Board*	−0.0382 (−0.1185)	−0.0584 (−0.1818)
*Top1*	−0.2213 (−0.5733)	−0.2025 (−0.5233)
*RD*	0.0621*** (3.8240)	0.0624*** (3.8720)
*Disclosure*	0.2976* (1.7692)	0.2864* (1.6819)
*Constant*	−10.1353*** (−9.2979)	−10.0843*** (−9.5599)
*Year*	*Control*	*Control*
*Industry*	*Control*	*Control*
*Observations*	2,355	2,355
*Pseudo R^2^*	0.1159	0.1178

The *T* value calculated after misadjustment of clustering standard at company level is in brackets; *, ** and *** are significant at the significance level of 10, 5 and 1%, respectively. The following table is the same.

The results in Table 8 show that after switching to the regression treatment, the results were still robust, the significance improved, and the regression coefficients were still positive. The results in columns (2) and (4) show that an increase in the degree of ESG disclosure (ESG_LN) on executives’ microblogs can significantly increase the number of green inventions and green utility models obtained by companies in the same year. This further indicates that an increase in the degree of ESG disclosure (ESG_LN) on executives’ microblogs can significantly increase the level of companies’ green innovation. The results in column (1) did not improve in significance, but the overall regression coefficient was still positive. The results in column (3) also passed the 1% level test on top of the positive coefficient. The above empirical results strongly support the hypothesis proposed in this paper, and the conclusions drawn are reliable and robust.

**Table 8:** Executives’ ESG cognition and green inventions.

Variables	(1)	(2)	(3)	(4)
	Green_Invention	Green_Invention	Green utility	Green utility
*ESG Ratio*	0.0038 (1.1737)		0.0096*** (3.4524)	
*ESG_LN*		0.0533** (2.0978)		0.0481** (2.4260)
*SOE*	−0.1624 (−0.9205)	−0.1513 (−0.8635)	−0.1546 (−1.0314)	−0.1551 (−1.0336)
*ROA*	3.8589*** (2.7202)	3.6627** (2.5643)	3.9749*** (2.9416)	3.8875*** (2.9065)
*LEV*	1.7717*** (3.4398)	1.6822*** (3.2921)	1.4218*** (3.6634)	1.3723*** (3.6063)
*SIZE*	1.2073*** (8.2668)	1.1930*** (8.4546)	0.8058*** (6.0498)	0.8049*** (6.1347)
*AGE*	0.0066 (0.0213)	−0.0306 (−0.0979)	−0.1775 (−0.7416)	−0.2084 (−0.8463)
*Board*	−0.2080 (−0.4730)	−0.2516 (−0.5791)	−0.0415 (−0.1272)	−0.0621 (−0.1896)
*Top1*	−0.3205 (−0.6618)	−0.2890 (−0.5922)	−0.3908 (−0.8543)	−0.3733 (−0.8216)
*RD*	0.0980*** (4.6677)	0.0972*** (4.6847)	0.0540*** (3.3550)	0.0550*** (3.4282)
*Disclosure*	0.4400* (1.9114)	0.4243* (1.8130)	0.2175 (1.1304)	0.2064 (1.0545)
*Constant*	−15.6772*** (−9.7139)	−15.4972*** (−9.9338)	−9.7628*** (−8.1247)	−9.7687*** (−8.3240)
*Year*	*Control*	*Control*	*Control*	*Control*
*Industry*	*Control*	*Control*	*Control*	*Control*
*N*	2,355	2,355	2,355	2,355
*Pseudo R^2^*	0.1392	0.1419	0.1290	0.1313

The *T* value calculated after misadjustment of clustering standard at company level is in brackets; *, ** and *** are significant at the significance level of 10, 5 and 1%, respectively. The following table is the same.

## Heterogeneity analysis

### Industry heterogeneity analysis

Table 9 reports the results of the regressions distinguishing between heavily polluting firms and non-polluting firms. In columns (2) and (4), the regression coefficients of the proportion of ESG disclosure (ESG_Ratio) and the degree of disclosure (ESG_LN) on executives’ microblogs were 0.0286 and 0.0359 respectively, both of which were significantly positive at the 1% level. The regression coefficients of the proportion of ESG disclosure (ESG_Ratio) and the degree of disclosure (ESG_LN) on executives’ microblogs in columns (1) and (3) were not significant. This indicates that non-heavily polluting firms are more efficient in green innovation compared to heavily polluting firms. Further, an increase in the proportion of ESG disclosure (ESG_Ratio) and the degree of disclosure (ESG_LN) on executives’ microblogs is also more likely to improve the level of firms’ green innovation.

**Table 9:** Industry heterogeneity analysis.

Variables	(1)	(2)	(3)	(4)
	Heavy pollution	Nonheavy pollution	Heavy pollution	Nonheavy pollution
*ESG Ratio*	−0.0050 (−0.4130)	0.0286*** (3.2027)		
*ESG_LN*			−0.0039 (−0.5082)	0.0359*** (3.1981)
*SOE*	−0.0013 (−0.0296)	−0.1954*** (−2.9374)	−0.0007 (−0.0156)	−0.1935*** (−2.9065)
*ROA*	1.5859*** (3.6976)	1.4240* (1.8616)	1.5755*** (3.6785)	1.3104* (1.7085)
*LEV*	0.3456*** (2.7920)	0.6724*** (3.5593)	0.3466*** (2.7991)	0.6536*** (3.4568)
*SIZE*	0.2162*** (4.8676)	0.9918*** (14.4688)	0.2175*** (4.8901)	0.9929*** (14.4901)
*AGE*	−0.2081*** (−3.6003)	0.0926 (1.2502)	−0.2080*** (−3.5985)	0.0771 (1.0385)
*Board*	0.1695* (1.7722)	−0.0833 (−0.5479)	0.1711* (1.7891)	−0.0775 (−0.5097)
*Top1*	0.2474* (1.9141)	−0.4180** (−2.1391)	0.2438* (1.8836)	−0.4124** (−2.1105)
*RD*	0.0057 (0.6773)	0.0406*** (5.7517)	0.0060 (0.7129)	0.0421*** (5.9572)
*Disclosure*	0.0160 (0.2387)	0.2383** (2.3641)	0.0168 (0.2503)	0.2346** (2.3270)
*Constant*	−2.2570*** (−5.0634)	−9.0604*** (−13.2073)	−2.2637*** (−5.0786)	−9.1377*** (−13.3505)
*Year*	*Control*	*Control*	*Control*	*Control*
*Industry*	*Control*	*Control*	*Control*	*Control*
*N*	1,018	1,337	1,018	1,337
*Adj_R^2^*	0.1047	0.2544	0.1047	0.2544
*p* value	0.0014		0.0428	

The *T* value calculated after misadjustment of clustering standard at company level is in brackets; *, ** and *** are significant at the significance level of 10, 5 and 1%, respectively. The following table is the same.

### Financing constraints

Corporate green innovation activities are influenced by financing constraints. To test the mechanism of the influence of executive ESG perceptions on corporate green innovation, we used the SA index to measure the level of financing constraints of firms and re-tested the sample by grouping them at the median of the SA index. Table 10 reports the regression results after grouping by the mean value of financing constraints. The results in columns (2) and (4) show that the regression coefficients for the proportion of ESG disclosure by executive microbloggs (ESG_Ratio) and the degree of disclosure (ESG_LN) were 0.030 and 0.022 and significant at the 1 and 5% test levels, respectively. Thus, the findings in Table 10 suggest that the impact of executive ESG perceptions on corporate green innovation is mainly found in firms with low financing constraints.

**Table 10:** Financing constraints heterogeneity analysis.

Variables	(1)	(2)	(3)	(4)
	Higher financing constraint	Lower financing constraint	Higher financing constraint	Lower financing constraint
*ESG Ratio*	0.0051 (0.2338)	0.0296*** (3.8050)		
*ESG_LN*			0.0313*** (2.7757)	0.0215** (2.1840)
*SOE*	0.0309 (0.5632)	−0.3165*** (−4.3781)	0.0384 (0.7019)	−0.3330*** (−4.5967)
*ROA*	0.8465 (1.3353)	2.3177*** (3.4721)	0.8366 (1.3243)	2.3620*** (3.5209)
*LEV*	0.5801*** (3.4307)	0.5502*** (3.0708)	0.5554*** (3.2967)	0.5432*** (3.0177)
*SIZE*	0.4211*** (6.2529)	0.7988*** (13.1642)	0.4058*** (6.0276)	0.8095*** (13.3111)
*AGE*	0.2236* (1.8390)	0.0922 (1.1745)	0.2132* (1.7618)	0.0918 (1.1621)
*Board*	−0.0293 (−0.2064)	0.0294 (0.2203)	−0.0459 (−0.3251)	0.0415 (0.3102)
*Top1*	−0.1409 (−0.7634)	−0.2342 (−1.3375)	−0.1208 (−0.6567)	−0.2243 (−1.2751)
*RD*	0.0387*** (4.7646)	0.0393*** (5.3600)	0.0383*** (4.7416)	0.0403*** (5.4759)
*Disclosure*	0.1951** (2.3148)	0.0283 (0.2697)	0.1873** (2.2293)	0.0240 (0.2278)
*Constant*	−5.4081*** (−5.2405)	−7.7835*** (−13.1648)	−5.2384*** (−5.0861)	−7.9484*** (−13.4307)
*Year*	*Control*	*Control*	*Control*	*Control*
*Industry*	*Control*	*Control*	*Control*	*Control*
*N*	1,106	1,249	1,106	1,249
*Adj_R^2^*	0.1589	0.2827	0.1648	0.2771
*p* value	0.4795		0.6947	

The *T* value calculated after misadjustment of clustering standard at company level is in brackets; *, ** and *** are significant at the significance level of 10, 5 and 1%, respectively. The following table is the same.

### Regional marketization process

When companies engage in green innovation, it is difficult to keep the pace and level of development of different companies synchronized, and there can be priorities or lags depending on various factors. There are differences in the level of social responsibility and technological innovation between firms in different marketization regions. Studies have shown that the degree of marketization has an impact on the level of social responsibility and innovation commitment of firms. Following Fang (2011), we used the regional marketization index score to measure the regional marketization process and used the median of the sample as the basis for grouping the regional marketization process.

Table 11 reports the results of regressions that grouped the means of the regional marketization process. A high marketization process was conducive to firm innovation in those regions. Our results from this grouped regression also mirror the above findings. The results in columns (1) and (3) show that the regression coefficients for the proportion of ESG disclosure by executive microbloggers (ESG_Ratio) and the degree of disclosure (ESG_LN) were 0.026 and 0.022 respectively, both significant at the 1% test level. This suggests that in regions with a higher marketization process, an increase in ESG awareness among executives would be more conducive to an increase in the level of corporate innovation.

**Table 11:** Group regression analysis of regional marketization process.

Variables	(1)	(2)	(3)	(4)
	High marketization process	Low marketization process	High marketization process	Low marketization process
*ESG Ratio*	0.0261*** (3.4609)	−0.0196 (−0.5159)		
*ESG_LN*			0.0218*** (2.5857)	0.0010 (0.0767)
*SOE*	−0.0625 (−1.1575)	−0.0950* (−1.6603)	−0.0684 (−1.2645)	−0.0959* (−1.6754)
*ROA*	1.6124*** (2.9181)	0.9139 (1.4646)	1.5970*** (2.8804)	0.9473 (1.5115)
*LEV*	0.5148*** (3.5100)	0.3475** (2.0726)	0.5055*** (3.4380)	0.3445** (2.0548)
*SIZE*	0.8342*** (15.5100)	0.1922*** (3.4221)	0.8367*** (15.5259)	0.1944*** (3.4684)
*AGE*	0.0276 (0.4628)	−0.2368*** (−2.7751)	0.0232 (0.3877)	−0.2364*** (−2.7682)
*Board*	−0.1086 (−0.9347)	0.2709** (2.0561)	−0.1080 (−0.9276)	0.2753** (2.0930)
*Top1*	−0.3549** (−2.3913)	0.2156 (1.1103)	−0.3511** (−2.3623)	0.2014 (1.0462)
*RD*	0.0465*** (7.1102)	−0.0022 (−0.3177)	0.0466*** (7.1052)	−0.0022 (−0.3122)
*Disclosure*	0.1181 (1.4627)	0.1001 (1.2016)	0.1174 (1.4525)	0.0974 (1.1674)
*Constant*	−7.6800*** (−14.5916)	−2.1682*** (−3.3951)	−7.7367*** (−14.6945)	−2.2062*** (−3.4582)
*Year*	*Control*	*Control*	*Control*	*Control*
*Industry*	*Control*	*Control*	*Control*	*Control*
*N*	1,877	478	1,877	478
*Adj_R^2^*	0.2591	0.1347	0.2570	0.1342
*p* value	0.1324		0.3369	

The *T* value calculated after misadjustment of clustering standard at company level is in brackets; *, ** and *** are significant at the significance level of 10, 5 and 1%, respectively. The following table is the same.

## Conclusion and discussion

According to cognitive theory, the behavior of corporate executives is governed and influenced by their cognition. On this basis, executive ESG cognition has a guiding effect on corporate green innovation behavior. Based on data from personal microblogs of executives of Chinese listed companies, we used text analysis to construct quantitative indicators of ESG perceptions of company executives. Further, using data from Chinese listed manufacturing companies, we empirically investigated the impact of executive ESG perceptions on corporate green innovation. The findings show that executive ESG perceptions significantly improve the level of corporate green innovation. Moreover, the findings of this paper still held after the endogeneity issue was addressed through a series of robustness tests. Further research found that the enhancing effect of executive ESG perception on the level of corporate green innovation was mainly found in the sample without heavy pollution and with lower financing constraints and a higher marketization process.

The research in this paper incorporates the cognitive factors of corporate executives into the study of corporate green innovation, further enriching and extending the study of the economic consequences of cognitive theory on corporate innovation behavior. It also further enriches the study of the influencing factors of corporate green innovation behavior from the perspective of managers’ cognition. In addition to this, the findings of this paper have important practical implications for improving corporate green innovation. At the enterprise level, enterprises should follow the general trend of green development, continuously enhance their own green awareness, and actively implement various green behaviors to achieve the development goal of a win-win situation for both the economy and the environment. On the one hand, enterprises should strengthen their ability to perceive resource and environmental issues, enrich their resource and environmental knowledge, raise their awareness of resource conservation and environmental protection responsibilities, and cultivate senior management’s awareness of green competitive advantages. ESG awareness is the spiritual source of green development and plays a vital role in enterprises’ acquisition of green performance. Enterprises should focus on the implementation of green behaviors, actively engage in green technological innovation, green production and other environmentally friendly behaviors, actively seek green development resources, and plan and allocate them appropriately. Enterprises should also accurately grasp policy dividends and maximize government funding support to broaden the resource base for green behaviors. At the government level, various incentive policies should continue to be used to drive the green performance of enterprises. Governments should continue to strengthen environmental regulations through strict environmental regulatory instruments, promote the establishment of ESG concepts among corporate executives, enhance ESG awareness, raise corporate awareness of green development, and implement green innovation strategies. However, the excessive costs and risks of green production, governance, and innovation have led to a lack of incentive for companies to acquire green performance. The government therefore needs to further improve various incentive policies by providing innovation subsidies and tax breaks for companies, which in turn will enhance their incentive to produce and innovate in a green way. The government should continue to strengthen market supervision, promote green consumption behavior, and boost the market’s awareness of the green development of enterprises, forming a mutual push between the green development of enterprises and the market.

This paper had some limitations that must be acknowledged. First, as this paper only selected companies in the manufacturing industry, where environmental pollution is a prominent issue and where executives have personal microblogs, the sample size was relatively small. Second, although this paper draws on existing research to define executive ESG perceptions, due to the subjective nature of the textual analysis and the extent of information disclosure on Weibo, the measurement of executive ESG perceptions may have inevitably been biased, and a more scientific measurement method should be adopted in the future in order to improve the reliability of research findings.

## Data availability statement

The original contributions presented in the study are included in the article/supplementary material, further inquiries can be directed to the corresponding author/s.

## Author contributions

DW, YL, SH, and QY have made important contributions. DW, YL, and SH involved in the idea construction of the paper, the data analysis and the first draft. QY involved in the data collected, the data analysis and the first draft. All authors contributed to the article and approved the submitted version.

## Funding

YL acknowledges financial support from the National Natural Science Foundation of China (grant number: 72002043). Shiyang Hu acknowledges financial support from the National Natural Science Foundation of China (grant numbers: 72272018 and 71802029) and the Fundamental Research Funds for the Central Universities (grant number: 2022CDJSKJC13).

## Conflict of interest

The authors declare that the research was conducted in the absence of any commercial or financial relationships that could be construed as a potential conflict of interest.

## Publisher’s note

All claims expressed in this article are solely those of the authors and do not necessarily represent those of their affiliated organizations, or those of the publisher, the editors and the reviewers. Any product that may be evaluated in this article, or claim that may be made by its manufacturer, is not guaranteed or endorsed by the publisher.

## References

[ref1] Aguilera-Caracuel J. Ortiz-de-Mandojana N. (2013). Green innovation and financial performance: an institutional approach. Organ. Environ. 26, 365–385. doi: 10.1177/1086026613507931

[ref2] Albuquerque R. Koskinen Y. Zhang C. (2019). Corporate social responsibility and firm risk: theory and empirical evidence. Manag. Sci. 65, 4451–4469. doi: 10.1287/mnsc.2018.3043

[ref3] Bandura A. (1989). Human agency in social cognitive theory. Am. Psychol. 44, 1175–1184. doi: 10.1037/0003-066X.44.9.1175 2782727

[ref4] Bandura A. (1991). Social cognitive theory of self-regulation. Organ. Behav. Hum. Decis. Process. 50, 248–287. doi: 10.1016/0749-5978(91)90022-L

[ref5] Beauchamp M. R. Crawford K. L. Jackson B. (2019). Social cognitive theory and physical activity: mechanisms of behavior change, critique, and legacy. Psychol. Sport Exerc. 42, 110–117. doi: 10.1016/j.psychsport.2018.11.009

[ref6] Berthet V. (2022). The impact of cognitive biases on professionals’ decision-making: a review of four occupational areas. Front. Psychol. 12:802439. doi: 10.3389/fpsyg.2021.802439 35058862 PMC8763848

[ref8] Chen Y. S. (2008). The driver of green innovation and green image-green core competence. J. Bus. Ethics 81, 531–543. doi: 10.1007/s10551-007-9522-1

[ref9] Chen S. S. Ho K. Y. Ho P. H. (2014). CEO overconfidence and long-term performance following R&D increases. Financ. Manag. 43, 245–269. doi: 10.1111/fima.12035

[ref10] Chen J. Liu L. (2020). Customer participation, and green product innovation in SMEs: the mediating role of opportunity recognition and exploitation. J. Bus. Res. 119, 151–162. doi: 10.1016/j.jbusres.2019.05.033

[ref11] Chen X. Yi N. Zhang L. Li D. (2018). Does institutional pressure foster corporate green innovation? Evidence from China’s top 100 companies. J. Clean. Prod. 188, 304–311. doi: 10.1016/j.jclepro.2018.03.257

[ref12] Cho T. S. Hambrick D. C. (2006). Attention as the mediator between top management team characteristics and strategic change: the case of airline deregulation. Organ. Sci. 17, 453–469. doi: 10.1287/orsc.1060.0192

[ref13] Cristofaro M. (2020). “I feel and think, therefore I am”: an affect-cognitive theory of management decisions. Eur. Manag. J. 38, 344–355. doi: 10.1016/j.emj.2019.09.003

[ref14] Delgado-Ceballos J. Aragón-Correa J. A. Ortiz-de-Mandojana N. Rueda-Manzanares A. (2012). The effect of internal barriers on the connection between stakeholder integration and proactive environmental strategies. J. Bus. Ethics 107, 281–293. doi: 10.1007/s10551-011-1039-y

[ref15] Eftekhar A. Fullwood C. Morris N. (2014). Capturing personality from Facebook photos and photo-related activities: how much exposure do you need? Comput. Hum. Behav. 37, 162–170. doi: 10.1016/j.chb.2014.04.048

[ref1004] Fang W. (2011). Market Development and Investment Strategies in Asia: Guest Editor’s Introduction. Emerg. Mark. Finance Trade 58, 3–5.

[ref16] Fernando Y. Wah W. X. (2017). The impact of eco-innovation drivers on environmental performance: empirical results from the green technology sector in Malaysia. Sustain. Prod. Consum. 12, 27–43. doi: 10.1016/j.spc.2017.05.002

[ref18] Gao P. Wang Y. Zou Y. Su X. Che X. Yang X. (2022). Green technology innovation and carbon emissions nexus in China: does industrial structure upgrading matter? Front. Psychol. 13:951172. doi: 10.3389/fpsyg.2022.951172, PMID: 35959076 PMC9362775

[ref19] Graf-Vlachy L. Bundy J. Hambrick D. C. (2020). Effects of an advancing tenure on CEO cognitive complexity. Organ. Sci. 31, 936–959. doi: 10.1287/orsc.2019.1336

[ref20] Gunasekaran A. Spalanzani A. (2012). Sustainability of manufacturing and services: investigations for research and applications. Int. J. Prod. Econ. 140, 35–47. doi: 10.1016/j.ijpe.2011.05.011

[ref21] Hambrick D. C. (2007). Upper echelons theory: an update. Acad. Manag. Rev. 32, 334–343. doi: 10.5465/amr.2007.24345254

[ref22] Hojnik J. Ruzzier M. (2016). The driving forces of process eco-innovation and its impact on performance: insights from Slovenia. J. Clean. Prod. 133, 812–825. doi: 10.1016/j.jclepro.2016.06.002

[ref24] Huang Q. Chen X. Zhou M. Zhang X. Duan L. (2019). How does CEO’s environmental awareness affect technological innovation? Int. J. Environ. Res. Public Health 16:261. doi: 10.3390/ijerph16020261, PMID: 30658493 PMC6352152

[ref25] Huang J. W. Li Y. H. (2017). Green innovation and performance: the view of organizational capability and social reciprocity. J. Bus. Ethics 145, 309–324. doi: 10.1007/s10551-015-2903-y

[ref1001] Kim M. Zhang L. Yu J. H. Koenigsfeld J. P. Cichy R. F. (2016). Private club GMs’/COOs perceptions in adopting social media: Applying the technology acceptance model. J. Hosp. Tour. Manag. 4, 37–48.

[ref26] Li H. Hang Y. Shah S. G. M. Akram A. Ozturk I. (2020). Demonstrating the impact of cognitive CEO on firms’ performance and CSR activity. Front. Psychol. 11:278. doi: 10.3389/fpsyg.2020.00278, PMID: 32184747 PMC7058782

[ref28] Lin Y. H. Chen Y. S. (2017). Determinants of green competitive advantage: the roles of green knowledge sharing, green dynamic capabilities, and green service innovation. Qual. Quant. 51, 1663–1685. doi: 10.1007/s11135-016-0358-6

[ref29] Liu Y. Xi M. (2021). Linking CEO entrepreneurial orientation to firm performance: the perspective of middle managers. Entrep. Theory Pract. 46:10422587211033571. doi: 10.1177/10422587211033571

[ref30] Liu X. Yang J. Qu S. Wang L. Shishime T. Bao C. (2012). Sustainable production: practices and determinant factors of green supply chain management of Chinese companies. Bus. Strategy Environ. 21, 1–16. doi: 10.1002/bse.705

[ref31] Mo X. Boadu F. Liu Y. Chen Z. Ofori A. S. (2022). Corporate social responsibility activities and green innovation performance in organizations: do managerial environmental concerns and green absorptive capacity matter? Front. Psychol. 13:13. doi: 10.3389/fpsyg.2022.938682 PMC9302574 35874421

[ref32] Obschonka M. Fisch C. (2018). Entrepreneurial personalities in political leadership. Small Bus. Econ. 50, 851–869. doi: 10.1007/s11187-017-9901-7

[ref33] Orens R. Reheul A. M. (2013). Do CEO demographics explain cash holdings in SMEs? Eur. Manag. J. 31, 549–563. doi: 10.1016/j.emj.2013.01.003

[ref1002] Ou Y. K. Lin C. H. Fang C. W. Liu Y. C. (2018). Using virtual environments to assess visual perception judgements in patients with Parkinson’s disease. Transp. Res. F: Traffic Psychol. Behav. 56, 322–332.

[ref1003] Park C. Kim Y. Jeong M. (2018). Influencing factors on risk perception of IoT-based home energy management services. Telemat. Inform. 35, 2355–2365.

[ref34] Pennebaker J. W. Mayne T. J. Francis M. E. (1997). Linguistic predictors of adaptive bereavement. J. Pers. Soc. Psychol. 72, 863–871. doi: 10.1037/0022-3514.72.4.863, PMID: 9108699

[ref35] Porter M. E. Van der Linde C. (1995). Toward a new conception of the environment–competitiveness relationship. J. Econ. Perspect. 9, 97–118. doi: 10.1257/jep.9.4.97

[ref36] Qiu L. Jie X. Wang Y. Zhao M. (2020). Green product innovation, green dynamic capability, and competitive advantage: evidence from Chinese manufacturing enterprises. Responsib. Environ. Manag. 27, 146–165. doi: 10.1002/csr.1780

[ref37] Sarfraz M. Ozturk I. Shah S. G. M. Maqbool A. (2020). Contemplating the impact of the moderators agency cost and number of supervisors on corporate sustainability under the aegis of a cognitive CEO. Front. Psychol. 11:965. doi: 10.3389/fpsyg.2020.00965, PMID: 32536890 PMC7267056

[ref38] Schunk D. H. DiBenedetto M. K. (2020). Motivation and social cognitive theory. Contemp. Educ. Psychol. 60:101832. doi: 10.1016/j.cedpsych.2019.101832

[ref40] She Z. Li Q. Zhou J. (2021). How CEO workaholism influences firm performance: The roles of collective organizational engagement and TMT power distance. Front Psychol. 12:725199. doi: 10.3389/fpsyg.2021.725199 34621222 PMC8490670

[ref41] Simon H. A. (1955). A behavioral model of rational choice. Q. J. Econ. 69, 99–118. doi: 10.2307/1884852

[ref42] Singh S. K. Del Giudice M. Chierici R. Graziano D. (2020). Green innovation and environmental performance: the role of green transformational leadership and green human resource management. Forecast. Soc. Change 150:119762. doi: 10.1016/j.techfore.2019.119762

[ref43] Sutcliffe K. M. Huber G. P. (1998). Firm and industry as determinants of executive perceptions of the environment. Strateg. Manag. J. 19, 793–807. doi: 10.1002/(SICI)1097-0266(199808)19:8<793::AID-SMJ980>3.0.CO;2-Y

[ref44] Tan Y. Zhu Z. (2022). ‘The effect of ESG rating events on corporate green innovation in China: the mediating role of financial constraints and managers’ environmental. Technol. Soc. 68:101906. doi: 10.1016/j.techsoc.2022.101906

[ref45] Tausczik Y. R. Pennebaker J. W. (2010). The psychological meaning of words: LIWC and computerized text analysis methods. J. Lang. Soc. Psychol. 29, 24–54. doi: 10.1177/0261927X09351676

[ref46] Wang H. Khan M. A. S. Anwar F. Shahzad F. Adu D. Murad M. (2021). Green innovation practices and its impacts on environmental and organizational performance. Front. Psychol. 11:553625. doi: 10.3389/fpsyg.2020.553625, PMID: 33536958 PMC7848084

[ref47] Wood R. Bandura A. (1989). Social cognitive theory of organizational management. Acad. Manag. Rev. 14, 361–384. doi: 10.2307/258173

[ref48] Xu N. Fan X. Hu R. (2022). Adoption of green industrial internet of things to improve organizational performance: the role of institutional isomorphism and green innovation practices. Front. Psychol. 13:917533. doi: 10.3389/fpsyg.2022.917533

[ref49] Yang Y. Hsu J. H. Löfgren K. Cho W. (2021). Cross-platform comparison of framed topics in twitter and Weibo: machine learning approaches to social media text mining. Soc. Netw. Anal. Min. 11, 1–18. doi: 10.1007/s13278-021-00772-w

[ref50] Zajac E. J. Bazerman M. H. (1991). Blind spots in industry and competitor analysis: implications of interfirm (mis) perceptions for strategic decisions. Acad. Manag. Rev. 16, 37–56. doi: 10.2307/258606

[ref51] Zhang L. Liang B. Bi D. Zhou Y. Yu X. (2021). Relationships among CEO narcissism, debt financing and firm innovation performance: emotion recognition using advanced artificial intelligence. Front. Psychol. 12:734777. doi: 10.3389/fpsyg.2021.734777 34589031 PMC8473637

[ref52] Zhang B. Wang Z. Lai K. H. (2015). Mediating effect of managers’ environmental concern: bridge between external pressures and firms’ practices of energy conservation in China. Environ. Psychol. 43, 203–215. doi: 10.1016/j.jenvp.2015.07.002

[ref53] Zheng J. Khurram M. U. Chen L. (2022). Can green innovation affect ESG ratings and financial performance? Evidence from Chinese GEM listed companies. Sustainability 14:8677. doi: 10.3390/su14148677

